# Supplementary material for: Extracellular DNA: A Critical Aspect of Marine Biofilms
Source: Microorganisms. 2022 Jun 24;10(7):1285. doi: 10.3390/microorganisms10071285 (PMC9320517; doi:10.3390/microorganisms10071285)
Supplement: Supplementary file 1 [file microorganisms-10-01285-s001.zip › microorganisms-1770989-supplementary.pdf]

microorganisms

SUPPLEMENTARY INFORMATION

Extracellular DNA a critical structural polymer in marine multi-species biofilms

Benjamin Tuck[a], Silvia Salgar-Chaparro[a], Elizabeth Watkin[b], Anthony Somers[c], Maria Forsyth[c] & Laura L. Machuca[a]*

[a]Curtin Corrosion Centre, WA School of Mines: Minerals, Energy and Chemical Engineering, Curtin University, Kent Street, Bentley, WA 6102, Australia. [b]Curtin Medical School, Curtin University, Kent Street, Bentley, WA 6102, Australia. [c]Institute for Frontier Materials, Deakin University, Burwood, VIC, 3217, Australia

Supplementary Table S1: ASW reactor solution composition used for all experiments. All reagents were AR grade.

Table S1: Artificial seawater solution	
Solution 1	g/L
Sodium Chloride (NaCl)	24.0
Sodium Sulfate (Na_2SO_4)	4.00
Potassium Chloride (KCl)	0.68
Potassium Bromide (KBr)	0.10
Boric Acid (H_3BO_3)	0.025
Sodium Fluoride (NaF)	0.002
Magnesium Chloride, hydrated ($MgCl_2*6H_2O$)	10.8
Calcium Chloride ($CaCl_2$)	0.10
Strontium Chloride ($SrCl_2$)	0.024
Bacto™ Casamino Acids	1.50
Sodium Pyruvate	0.55
Glucose	0.90

Ammonium Nitrate (NH_4NO_3)	1.20
Deionized water	968 mL
Solution 2	
Sodium Dihydrogen Phosphate (NaH_2PO_4)	0.04
Deionized water	10 mL
Solution 3	
Sodium Hydrogen Carbonate ($NaHCO_3$)	0.20
Deionized water	10 mL
Solution 4	
Ammonium Chloride (NH_4Cl)	0.50
Deionized water	10 mL
Solution 5	
Wolfe's Vitamins Solution*	1 mL
Trace Elements Solution*	1 mL
*Separate solution prepared directly as outlined in previous work[31]	

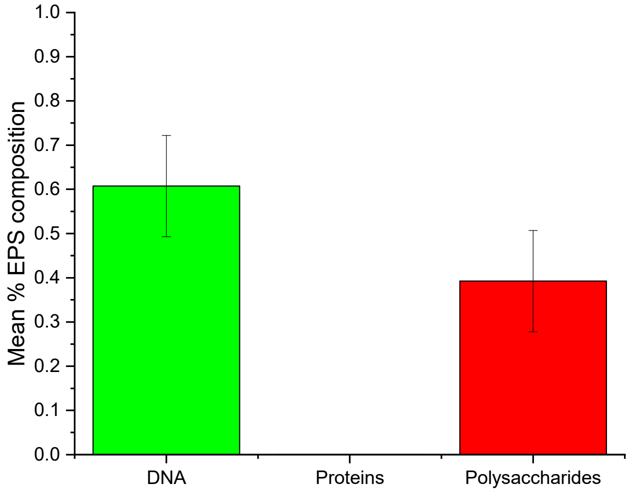

Supplementary Figure S1: The mean percent composition of eDNA, proteins and polysaccharides in the biofilm EPS matrix of reactor 2 at 2 weeks. Error bars represent the standard deviation of triplicate 20 x micrographs.

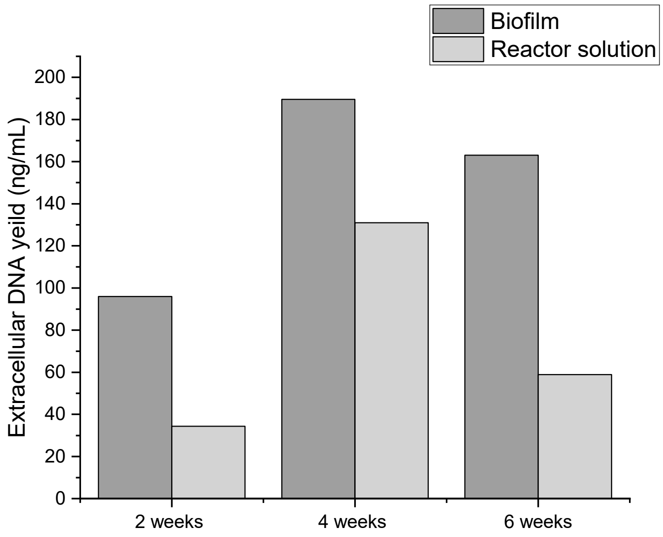

Supplementary Figure S2: Qubit quantification of eDNA from biofilms and reactor solution at 2, 4 and 6 weeks. Measurements were taken using a pooled sample from experimental replicates.

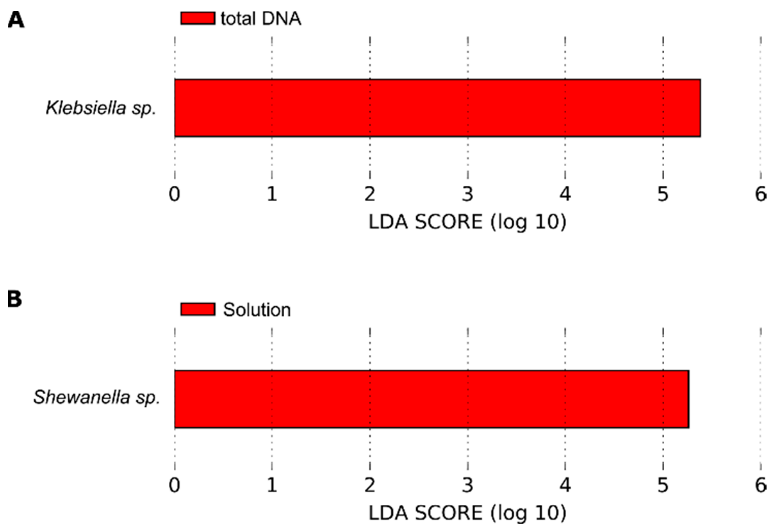

Supplementary Figure S3. Linear discriminant analysis effect size analysis LEfSe plot of taxonomic biomarkers. (A) Microbial genera significantly associated with DNA source. (B) Microbial genera significantly associated with microbial cell. Horizontal bars represent the effect size for each significantly associated taxon. The length of the bar represents the log10 transformed LDA score, indicated by vertical dotted lines.

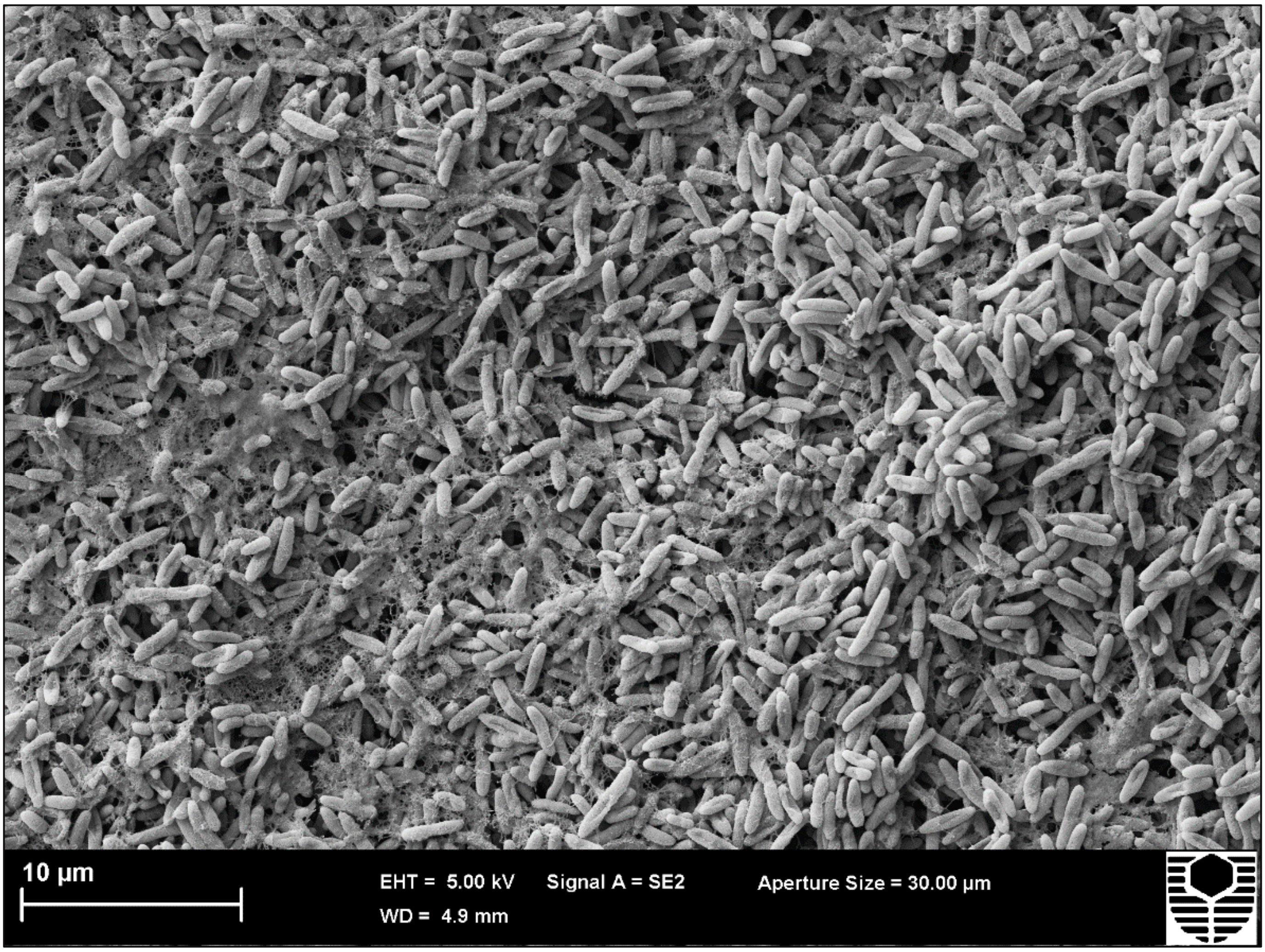

Supplementary Figure S4: SEM of *Pseudomonas balearica* EC28 biofilm grown on CS over 24 h. Surface coverage was 100%, and the strain revealed a fibrous, net-like EPS structure resembling eDNA.
